# The effect of diet and host genotype on ceca microbiota of Japanese quail fed a cholesterol enriched diet

**DOI:** 10.3389/fmicb.2015.01092

**Published:** 2015-10-07

**Authors:** Shasha Liu, Darin C. Bennett, Hein M. Tun, Ji-Eun Kim, Kimberly M. Cheng, Hongfu Zhang, Frederick C. Leung

**Affiliations:** ^1^The State Key Laboratory of Animal Nutrition, Institute of Animal Sciences, Chinese Academy of Agricultural SciencesBeijing, China; ^2^Faculty of Land and Food Systems, Avian Research Centre, The University of British ColumbiaVancouver, BC, Canada; ^3^School of Biological Sciences, The University of Hong KongHong Kong, Hong Kong

**Keywords:** quail model, cecal microbiome, cholesterol, atherosclerosis, genotype by diet interaction

## Abstract

Two Japanese quail strains, respectively atherosclerosis-susceptible (SUS) and –resistant (RES), have been shown to be good models to study cholesterol metabolism and transportation associated with atherosclerosis. Our objective was to examine possible difference in cecal microbiota between these strains when fed a control diet and a cholesterol enriched diet, to determine how host genotype and diet could affect the cecal microbiome that may play a part in cholesterol metabolism. A factorial study with both strains and two diets (control, cholesterol) was carried out. Cecal content was collected from 12 week old quail that have been on their respective diets for 6 weeks. DNA was extracted from the samples and the variable region 3–5 of the bacterial 16S rRNA gene was amplified. The amplicon libraries were subjected to pyrosequencing. Principal Component Analysis (PCA) of β-diversity showed four distinct microbiota communities that can be assigned to the 4 treatment groups (RES/control, RES/cholesterol, SUS/control, SUS/cholesterol). At the Phylum level, the 4 treatment groups has distinct *Firmicutes* community characteristics but no significant difference in *Bacteroidetes*. *Eubacterium dolichum* was rare in RES/control but became overabundant in RES/cholesterol. An unclassified species of *Lactobacillaceae* was found in abundance in SUS/control but the same species was rare in RES/cholesterol. On the other hand, two *Lactobacillus* species were only found in RES/control and an unclassified *Lachnospiraceae* species was abundant in RES/cholesterol but rare in SUS/control. The abundance of four species of *Lachnospiraceae*, three species of *Ruminococcaceae* and one species of *Coprobacillaceae* was positively correlated with plasma Total Cholesterol, plasma LDL, and LDL/HDL ratio. Our study of cecal microbiota in these quail has demonstrated that selection for susceptibility/resistance to diet induced atherosclerosis has also affected the quail's cecal environment to host distinctly different cecal microbiome.

## Introduction

Despite many measures are available for the management of cardiovascular disorders, this common disease is still associated with high incidence of mortality and morbidity (Go et al., 2014). This is a reflection that atherosclerosis is a complex pathological process, affected by both genetic and environmental factors that we still do not fully understand. Over the past several years, researchers have turned their attention to the effects of gastro-intestinal microbiota on the development of metabolic diseases (Cani and Delzenne, 2009; Caesar et al., 2010; Sekirov et al., 2010; Karlsson et al., 2012; Strowig et al., 2012). In particular, Wang et al. (2011) found that in mice, intestinal microbiota metabolism of dietary choline produced trimethylamine (TMA). TMA is further converted to trimethylamine N-oxide (TMAO) by liver enzymes Flavin monooxygenases, and TMAO has been found to promote endogenous cholesterol-laden macrophage foam cell formation, one of the earliest hallmarks of the atherosclerotic process. Koeth et al. (2013) found that L-carnitine, a TMA abundantly found in red meat, could also be converted to TMAO by intestinal microbiota. They also found that TMAO significantly reduced reverse cholesterol transport in mice. Never-the-less, most of the studies carried out so far lacked defined host genotypes and defined diets at the same time to examine their interaction effect on gut flora diversity.

The Japanese quail was introduced as a laboratory animal in the 1960s (Padgett and Ivey, 1959) and proved to be useful in many areas of biomedical studies (Minvielle et al., 2007; Cheng et al., 2010). At the University of British Columbia (UBC) Avian Research Centre, we have been maintaining two related strains of Japanese quail for research in atherosclerosis. The two strains were developed by divergent selection from a common foundation population (Shih et al., 1983). One strain is susceptible to diet-induced atherosclerosis (SUS), and the other is resistant (RES). When challenged with a high cholesterol diet, about 80% of the SUS males will develop atherosclerosis whereas only about 4% of the RES males will. Li et al. (2012) examined differential mRNA expression of seven genes involved in cholesterol metabolism and transport in the liver of the SUS and RES and concluded that these quail are good models for studying cholesterol metabolism and transport in relationship to atherosclerosis development. We therefore think that this quail model would be useful for studying the interaction of host genotype and diet in affecting the gut flora diversity in association with the development of atherosclerosis.

The objectives of our study were (1) to characterize the phylogenetic diversity of the cecal microbiota of the SUS and RES males fed a regular (control) diet, using 454 pyrosequencing after amplification for V3–V5 region of bacterial 16S rRNA gene, (2) to characterize the cecal microbiota diversity of the SUS and RES males fed the control diet dosed with cholesterol (0.5% w/w) using the same pyrosequencing procedure, and (3) to identify gut bacteria that are key to each of the four treatment groups (SUS/control diet, RES/control diet, SUS/cholesterol diet, RES/cholesterol diet) and to examine the association of these bacteria with the development of atherosclerosis.

## Materials and methods

### Experimental birds

The two strains of Japanese quail, SUS and RES, have been acquired by the UBC Quail Genetic Resource Centre from North Carolina State University in 1989. The history of their selective breeding has been described by Shih et al. (1983). Since their transfer to UBC, they have undergone further divergent selection for susceptibility and resistance to atherosclerotic plaque formation induced by dietary cholesterol (0.5%w/w) (Cheng et al., 1997).

### Experimental design

After hatching, both SUS (*N* = 80) and RES (*N* = 80) males were fed a semi-synthetic diet (Li et al., 2012) (Table 1) prepared by the feed mill at the Agriculture and Agri-Food Canada Poultry Research Station at Agassiz, British Columbia, according to the NRC nutrient requirements standards recommended for Japanese quail (http://www.nap.edu/catalog/2114.html). At 6 weeks of age, they were divided into two dietary treatment groups and fed either a regular synthetic diet (control) or a synthetic diet with added cholesterol (0.5%w/w) for another 6 weeks (Li et al., 2012). Individually marked birds (both RES and SUS) fed the same diet were kept in the same pen. Birds on the alternative diet were kept in a neighboring pen. At 12 weeks of age, 6 birds from each of the treatment groups with body weight closest to the mean of the population were euthanized by decapitation and trunk blood was collected into Vacutainer tubes (Becton–Dickinson, Mississauga, ON, Canada), containing lithium heparin, and centrifuged at 4°C for 10 min at 3000 × g. Plasma was stored at −20°C until it was later used for lipid analysis. Sections of ceca, including gut content were collected from each bird. All samples were quick frozen on dry ice immediately after collection and stored at −70°C until processed for DNA extraction. The aortic tree (the brachycephalic arteries to their bifurcations and the aorta to the iliac branching) of each bird was dissected out, opened longitudinally and examined under a 10–30X dissecting microscope for a semi-quantitative scoring of the seriousness of the atherosclerotic lesions on the interior wall. The scoring system was adopted from Godin et al. (1995). A score of 0 (normal) to 4 (presence of severe atherosclerotic lesions) was assigned by two independent scorers who were blind to the genetic and diet status of the bird. Four quail from each treatment group were selected, based on their atherosclerotic lesion scores, for examination of their cecal microbiota. This research was carried out with the approval of the UBC Animal Care Committee (Certificate # A12-0087).

**Table 1:** **Semi-synthetic diets**.

**Ingredients (g/kg)**	**Control diet**	**Cholesterol diet**
Soy protein flour (50% protein)	340.0	340.0
Corn starch	400.0	390.0
Limestone	50.0	50.0
Mineral premix	5.0	5.0
Monofos	30.0	30.0
Sucrose	20.0	20.0
Alphacel	70.0	70.0
Vitamin premix	5.0	5.0
D-L methionine	4.0	4.0
Choline chloride	3.8	3.8
Tallow	50.0	50.0
Vegetable oil	30.0	30.0
Cholesterol	0.0	5.0
Cholic acid	0.0	2.5

### Plasma lipid analysis

Plasma samples were sent to the Department of Pathology and Laboratory Medicine at St. Paul's Hospital (Vancouver, BC) and assayed for total cholesterol, HDL, and triglycerides using enzymatic methods on an ADVIA 1650 Chemistry System. Any sample with 3+ lipemia or greater (as measured by the analyzer), was cleared by Airfuge® Air-Driven ultracentrifugation (Beckman Coulter). Lipemia at that level can cause interference with the HDL assay. HDL was assessed by the direct method without precipitation of apolipoprotein B (Warnick and Albers, 1978; Warnick et al., 2001; Gootjes et al., 2009). LDL values were calculated by Friedewald's formula, using measured values for total cholesterol, HDL and triglycerides (Friedewald et al., 1972; Okada et al., 1998).

### DNA extraction and pyrosequencing

The intestinal segments were thawed and the contents were gently scraped from the intestinal wall. The surgical tools and vials were autoclaved and the bench area was wiped clean with 70% ethanol to minimize contamination. Genomic DNA was isolated using the PowerMax Soil DNA Isolation Kit (Mo. Bio laboratories. Inc., Carlsbad, CA) according to the instructions of the manufacturer with 200 mg as starting material. PCR amplifications were performed using the FastStart high fidelity PCR system (Roche Molecular Diagnostics, Branchburg, NJ, USA). The variable region 3–5 (V3–V5) of the bacterial 16S rRNA gene was amplified with a primer set of 341F (5′- ACTCCTACGG GAGGCAGCAG-3′) and 926R (5′- CCGTCAATTCMTTTGAGTTT-3′) with the sample specific forward primer bearing a multiplex identifier (MID) sequences. All 341F and 926R primers modified with adaptor A and B sequences respectively for pyrotag sequencing. The amplification program consisted of an initial denaturation step at 94°C for 2 min; 32 cycles of denaturation at 94°C for 30 s, annealing at 60°C for 30 s, and elongation at 72°C for 30 s; and a final extension step at 72°C for 7 min. The size of the PCR products was confirmed by gel electrophoresis. The PCR products was then purified using Gel extraction kit (Invitrogen) and were quantified using the NanoDrop 2000 (Thermo Scientific, Wilmington, DE, USA). The Amplicon libraries were subjected to pyrotag sequencing using a bench-top 454 GS Junior (454 Life Sciences-a Roche Company, Branford, CT, USA) with the GS Junior Titanium Sequencing Kit (https://lifescience.roche.com/shop/en/us/products/gs-junior-titanium-sequencing-kit).

### Sequence analysis

Sequences obtained from pyrosequencing were processed using the QIIME (quantitative insights into microbial ecology) software package (Caporaso et al., 2010b). Quality trimming of dataset removed sequences if a mean quality score was ≤ 25; lengths were < 150 or >900 bp; sequences were without primer, uncorrectable, or contained ambiguous characters; or homopolymer run exceeding 8 nt. De-noising of dataset was performed using DENOISER v. 0.9.1 (Quince et al., 2011) as implemented in QIIME platform. Chimeric sequences were removed using Chimera Slayer. The sequences were assigned to groups basing on their respective barcode sequences. Similar sequences were assigned into operational taxonomic units (OTUs) at a pairwise identify of 97% using UCLUST (http://www.drive5.com/usearch/). Representative sequence was the most abundant sequence in each OTU. Representative sequences (at 97% similarity) were then classified taxonomically using Ribosomal Database Project (RDP) classifier 2.0.1 (Cole et al., 2009). The OTUs were aligned using PyNAST with a minimum alignment length of 150 bp and a minimum percent identity of 75% (Caporaso et al., 2010a). After alignment, PH LANE mask (http://greengenes.lbl.gov/) was conducted to screen out the hypervariable regions.

### Statistical analysis

#### Richness and diversity indices

Rarefaction plots were constructed and diversity indices (Chao1 richness, Simpson's Diversity) were estimated as implemented in QIIME (Caporaso et al., 2010b). For the comparison of β-diversity among microbial communities, we used principal component analysis (PCA) to visualize all OTUs and OTUs in phylum level (*Firmicutes*) differences. Results of the PCA were then statistically tested by permutational multivariate analysis (PERMANOVA) of variance (Anderson et al., 2008) for ceca microbiota compositions differences among four treatment groups. Mahalanobis distance (de Maesschalck et al., 2000) was calculated to confirm the difference between every two cluster.

#### Comparison of microbial communities

Bacterial abundance difference on phylum-, family-, and species- levels were examined using multivariate analysis, and further using Tukey's HSD for mean separation (SPSS 13.0; SPSS Institute, 2001) and expressed as means ± SE.

Least squares analysis of variance was performed to compare the diversity and richness parameters using JMP 8.0 (SAS Institute, North Carolina, 2008). The statistical model as follow:

$$\begin{array}{l} {\text{Y}}_{\text{ijk}}=\mu +{\text{S}}_{\text{i}}+{\text{D}}_{\text{j}}+{\left(\text{SD}\right)}_{\text{ij}}+{\text{E}}_{\text{ijk}} \end{array}$$

Where Y_ijk_ represents the measure for the kth individual of the ith strain from jth diet; S_i_ = RES or SUS bird; D_j_ = control or a cholesterol diet; (SD_ij_) = the two-way interaction term and E_*ijk*_ = the error term. The results were reported as the least square mean ± standard error of means (SEM). Tukey's HSD was used for mean separation and statistical significance was defined at *P* < 0.05.

#### Key microbiota in each treatment group

We used Venn Diagram (Oliveros, 2007) and a “Nearest-shrunken Centroid” (NSC) classification approach (Tibshirani et al., 2002; Koren et al., 2011) to detect core microbiota community which best characterize each group. The amount of shrinkage was determined by cross-validation and test error was minimized. We selected the OTUs from all 6 Phyla that are common in at least 3 of 4 samples examined per treatment, and with a minimum sequence count of 100 sequences per OTU to generate a filtered OTU table for the Venn diagram and NSC analyses. NSC analysis was performed on normalized Z-score profiles of OTUs. The misclassification error was 0.25 and threshold was 1.13.

#### Correlation of abundance of OTUs with blood lipid parameters

A correlation heatmap was generated to examine the correlation between the abundance of particular OTUs with the levels of blood lipid parameters—plasma Total Cholesterol, plasma LDL, plasma HDL, plasma Triglycerides, and LDL/HDL ratio. Because the levels of blood lipid parameters were not independent of dietary cholesterol, only RE and SE were included in the analysis. Because of small sample size, correlation plots of significant correlations were examined for data point distribution to eliminate correlations due to outliers.

## Results

### Atherosclerotic lesions on the intimal surface of the aortae

All SUS and RES fed the control diet scored 0. All four SUS on cholesterol diet scored 4, while two RES on the same diet score 0 and two scored 1.

### Richness of ceca microbiota

After trimming, assembly and quality filtering, a total of 257,860 sequence reads were obtained with a mean reading length of 545 bp and 16,116 ± 4269 reads/sample. There was no significant difference among the four treatment groups in the number of sequence reads.

**Figure 1:**
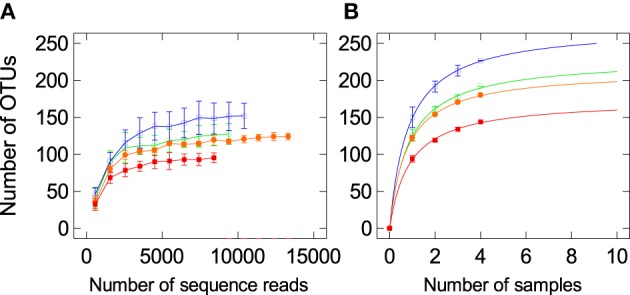
**Rarefaction analysis, calculated at 97% dissimilarity, for the assessment of operational taxonomic unit (OTU) coverage within the16S rRNA gene-based cecal bacterial communities in the RES and SUS quail fed the control (RC, blue open square; SC, green open square) or cholesterol (RE, orange closed square; SE, red closed square) diets**. **(A)** The number of OTUs as a function of the number of sequence reads. **(B)** The number of OTUs as a function of the number of individual quail sampled.

Rarefaction curves (Figure 1A) of the quality filtered sequences showed that our sequencing was deep enough to recover almost all of the OTUs in the sampled population [RES/control diet (RC) 150.0 ± 25.1 OTUs/sample, SUS/control diet (SC) 125.8 ± 14.4 OTUs /sample, RES/cholesterol diet (RE) 94.0 ± 8.5 OTUs /sample, SUS/cholesterol diet (SE) 122.2 ± 6.9 OTUs /sample]. Rarefaction curves (Figure 1B) also indicated that four samples per treatment group was a big enough sample size to detect most of the core OTUs in the population (Hughes and Hellmann, 2005).

The sequences were classifiable into 366 species-level operational taxonomic units (OTUs) (123 ± 24.7 OTUs/sample) belonging to 6 bacterial phyla. The vast majority (98%) of the sequences belonged to two bacterial phyla: *Firmicutes* (77%) and *Bacteroidetes* (21%).

The remaining sequences were identified as *Spirochaetes, Tenericutes, Proteobacteria*, and *Actinobacteria.* Because of very low number of sequences, we did not compare bacterial abundance differences in these phyla. The sequences have been submitted to Sequence Read Archive (SRE) with accession number SRR2537231.

### A comparison of ceca microbiota diversity

#### Community level variations

Chao1 estimator indicated that there was a significant (*P* = 0.002) diet × genotype interaction affecting richness. RES on the control diet (RC) had significantly higher OTU richness (164 ± 11.7) than RES on the cholesterol diet (RE: 104 ± 2.5), whereas there was no significant difference in richness between the two SUS dietary groups (SC: 138 ± 7.2, SE; 133 ± 2.1). There was no significant difference (*P* > 0.05) in diversity among the 4 treatment groups according to the Simpson estimate of diversity.

**Figure 2:**
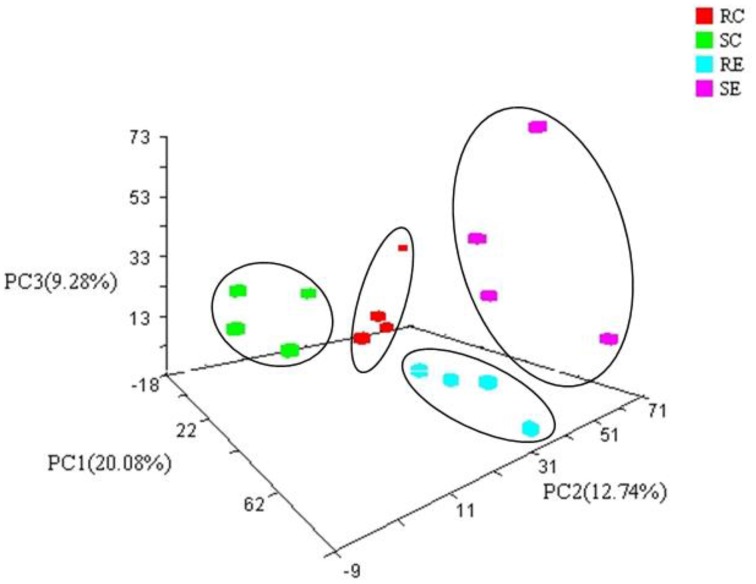
**Three-dimensional projection of PCA of whole cecal microbial community**. The variance explained by the PCs is indicated in parentheses on the axes. Each symbol represents a single sample.

**Figure 3:**
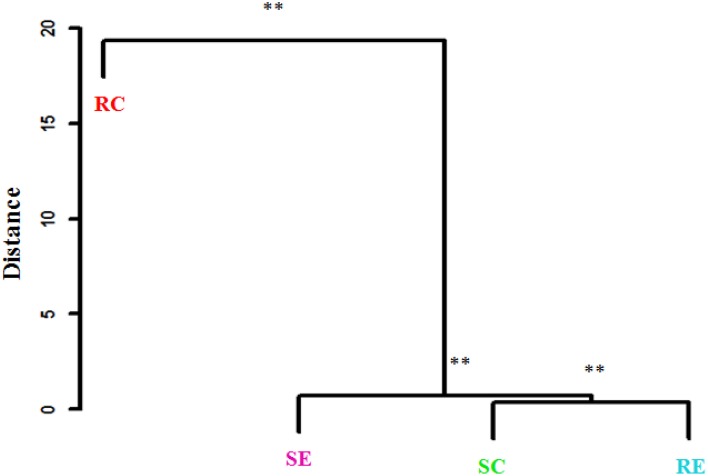
**Clustering of gut microbiota based on distances between different groups calculated with multivariate analysis of variance test of the first six PCs of OTUs data**. The Mahalanobis distances between group means are shown. ^**^P < 0.01.

Principal components Analysis (PCA) of β-diversity showed four distinct microbiota communities that can be assigned to the four treatment groups (Figures 2, 3). There was a significant (PERMANOVA; *P* = 0.02) diet × genotype interaction indicating these four treatment groups had distinct microbial community characteristics.

#### Phylum level variations

**Figure 4:**
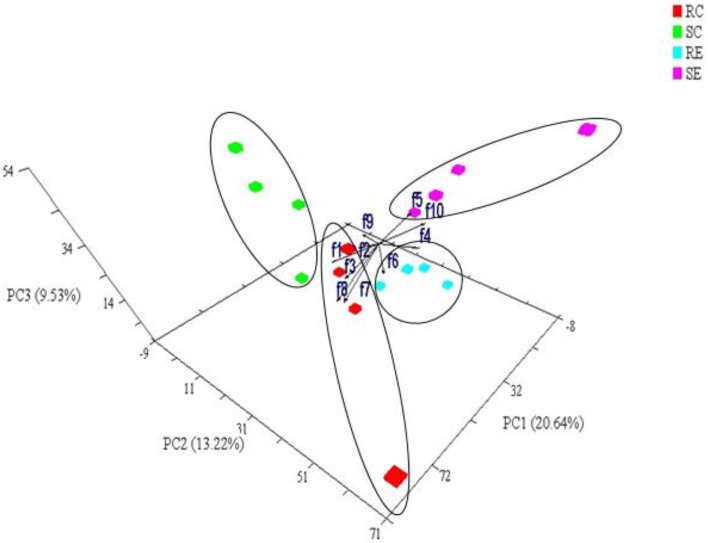
**Joint Plot of PCA of Phylum *Firmicutes* in cecal content**. The variance explained by the PCs is indicated in parentheses on the axes. Each symbol represents a single sample. The vector projections showed the relationship between family-level variables in *Firmicutes* and four treatment groups. The angle and length of the arrows indicated the direction and strength of the relationship. f1, Bacilli Lactobacillales Lactobacillaceae; f2, Bacilli Lactobacillales Streptococcaceae; f3, Unclassified *Bacilli*; f4, Clostridia Clostridiales Clostridiaceae; f5, Clostridia Clostridiales Lachnospiraceae; f6, Clostridia Clostridiales; f7, Unclassified Clostridia Clostridiales; f8, Unclassified *Clostridia*; f9, Erysipelotrichi Erysipelotrichales Coprobacillaceae; f10, Erysipelotrichi Erysipelotrichales Erysipelotrichaceae.

**Figure 5:**
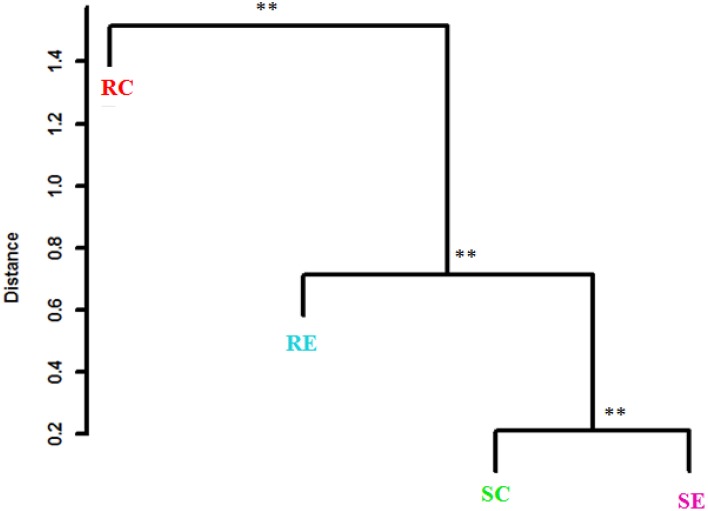
**Clustering of *Firmicutes* based on distances between different groups calculated with multivariate analysis of variance test of the first six PCs of OTUs data**. The Mahalanobis distances between group means are shown. ^**^P < 0.01.

PCA was also conducted for OTUs within a Phylum. Figure 4 showed the PCA of *Firmicutes* OTUs. There was a significant (PERMANOVA; *P* = 0.02) diet × genotype interaction indicating these four treatment groups had distinct *Firmicutes* community characteristics. Figure 5 showed the Mahalanobis distance between the groups to confirm their clustering. All *Bacteroidetes* belonged to the family *Rikenellaceae*. There was no significant difference among the treatment groups when OTUs within *Bacteroidetes* were analyzed.

#### Family level variations

We used Joint Plot of PCA (PC-ORD) to examine the correlation of microbiota families (within *Firmicutes*) with the four treatment groups (indicated by the arrows in Figure 4). In general, birds on control diet were characterized by the abundance of *Lactobacillaceae* and *Streptococcaceae*. Birds on cholesterol diet were characterized by the abundance of *Erysipelotrichaceae* and *Clostridiaceae*. Specifically, cecal microbiota in RC was characterized by the relative abundance of unclassified *Clostridiales, Bacilli* and *Clostridia*, and SC was characterized by *Coprobacillaceae*. SE was characterized by *Lachnospiraceae*, while RE by *Erysipelotrichaceae*.

Using multivariate analysis, we found a significant (*P* < 0.012) diet × genotype interaction in affecting the abundance of *Ruminococcaceae* (Table 2). RC had significantly more than RE, but there was no significant difference for SUS on the two different diets. The abundance of *Erysipelotrichaceae*, unclassified family of *Bacilli* (UB), and unclassified family of *Clostridia* (UC) was significantly affected by diet. Compared to birds (SUS and RES) on control diet, birds on cholesterol diet had significantly more *Erysipelotrichaceae*, significantly less UB (*P* < 0.04) and UC (*P* < 0.03).

**Table 2:** **Family level differences in abundance among treatment groups**.

**Microbiota**	**Treatment group**			
	**RC**	**SC**	**RE**	**SE**
*Ruminococcaceae*§	1722.0 ± 742.6^a^	932.0 ± 269.1^ab^	585.75 ± 242.3^b^	1179.2 ± 429.4^ab^
Erysipelotrichaceae^*^	110.4 ± 52.30^a^		291.6 ± 189.6^b^	
Uncl. Clostridia^*^	246.2 ± 213.0^a^		74.4 ± 38.1^b^	
Uncl. Bacilli^*^	48.5 ± 54.6^a^		7.7 ± 10.0^b^	
Uncl. Streptococcaceae^**^	90.5 ± 34.6		14.3 ± 8.1	

§ Significant diet × host genotype interaction;

* Significant diet effect;

** Effect of diet tends to be significant (P < 0.055).

In each row, means followed by different letter superscripts are significantly different by Tukey's HSD.

#### Genus level variations

Using multivariate analysis, we found a significant (*P* < 0.0001) diet effect in the abundance of *Ruminococcus* (Table 3) (Control diet: 125.63 ± 17.88, Cholesterol diet: 16.13 ± 15.36). We have also found a significant (*P* < 0.002) diet effect in the abundance of *Cc_115* (belonging to Family *Erysipelotrichaceae*). There was also a significant (*P* < 0.05) diet × genotype interaction in Unclassified *Ruminococcaceae* (Table 3).

**Table 3:** **Genus level differences in abundance among treatment groups**.

**Microbiota^‡^**	**Treatment group**			
	**RC**	**SC**	**RE**	**SE**
Uncl. *Ruminococcaceae*^§^	1202.75 ± 195.43^a^	710 ± 76.25^ab^	543.25 ± 113.60^b^	1123.25 ± 197.10^ab^
Uncl. Cc_115^*^	14.88 ± 2.29^a^		71.50 ± 12.69^b^	
Ruminococcus^*^	125.63 ± 17.88^a^		16.13 ± 5.43^b^	

‡ See Table 2 for Uncl. Clostridia, Uncl. Bacilli, and Uncl. Streptococcaceae.

§ Significant diet × host genotype interaction;

* Significant diet effect.

In each row, means followed by different letter superscripts are significantly different by Tukey's HSD.

**Figure 6:**
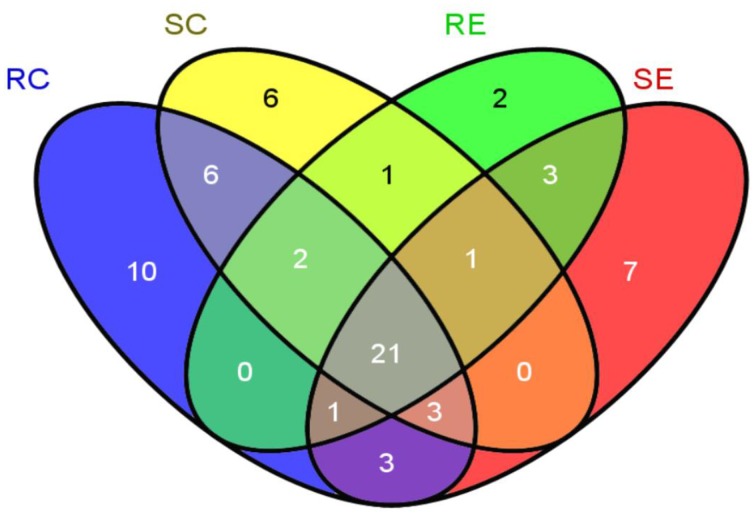
**Venn diagram indication of 66 OTUs identified in the four treatment groups**.

Venn diagram showed the distribution of 66 filtered OTUs (Figure 6). With much overlap, birds on the control diet harbored 54 OTUs while birds on the cholesterol diet harbored 44 OTUs. Twelve OTUs were unique to birds on the cholesterol diet, while 22 OTUs were unique to birds on the control diet (Table 4). Twelve OTUs were unique to RES birds and 13 were unique to SUS birds. Ten OTUs (7.36%) were unique to RC, 6 (1.57%) unique to SC, 2 (0.71%) to RE, and 7 (2.36%) to SE.

**Table 4:** **Unique OTUs generated by Venn diagram**.

	**ID**	**Phylum**	**Class**	**Order**	**Family**	**Genus**	**Species**
RC	1028036	*Firmicutes*	*Bacilli*	Unclassified			
	137580	*Firmicutes*	*Bacilli*	*Lactobacillales*	*Lactobacillaceae*	*Lactobacillus*	Unclassified
	137043	*Firmicutes*	*Bacilli*	*Lactobacillales*	*Lactobacillaceae*	*Lactobacillus*	*reuteri*
	195728	*Firmicutes*	*Clostridia*	Unclassified			
	NCUR 2	*Firmicutes*	*Clostridia*	*Clostridiales*		Unclassified	
	157479	*Firmicutes*	*Clostridia*	*Clostridiales*		Unclassified	
	40164	*Firmicutes*	*Clostridia*	*Clostridiales*		Unclassified	
	214684	*Firmicutes*	*Clostridia*	*Clostridiales*		Unclassified	
	1983368	*Firmicutes*	*Clostridia*	*Clostridiales*		*Anaerotruncus*	Unclassified
	519763	*Firmicutes*	*Clostridia*	*Clostridiales*		*Oscillospira*	Unclassified
SC	188057	*Firmicutes*	*Clostridia*	Unclassified			
	194417	*Firmicutes*	*Clostridia*	*Clostridiales*	*Lachnospiraceae*	Unclassified	
	186881	*Firmicutes*	*Clostridia*	*Clostridiales*		Unclassified	
	158217	*Firmicutes*	*Clostridia*	*Clostridiales*		Unclassified	
	366143	*Firmicutes*	*Clostridia*	*Clostridiales*		*Oscillospira*	Unclassified
	174654	*Firmicutes*	*Clostridia*	*Clostridiales*		*Ruminococcus*	*bromii*
RE	183867	*Firmicutes*	*Clostridia*	*Clostridiales*	*Lachnospiraceae*	Unclassified	
	2182669	*Firmicutes*	*Clostridia*	*Clostridiales*	*Lachnospiraceae*	Unclassified	
SE	146086	*Firmicutes*	*Clostridia*	*Clostridiales*	*Lachnospiraceae*	Unclassified	
	583089	*Firmicutes*	*Clostridia*	*Clostridiales*	*Lachnospiraceae*	*Blautia*	
	174695	*Firmicutes*	*Clostridia*	*Clostridiales*	*Lachnospiraceae*	*Ruminococcus*	Unclassified
	229097	*Firmicutes*	*Clostridia*	*Clostridiales*	*Lachnospiraceae*	*Ruminococcus*	Unclassified
	228232	*Firmicutes*	*Clostridia*	*Clostridiales*		Unclassified	
	189309	*Firmicutes*	*Clostridia*	*Clostridiales*		Unclassified	
	566391	*Firmicutes*	*Clostridia*	*Clostridiales*		Unclassified	
OTUs common to RC and SC	255359	*Firmicutes*	*Bacilli*	*Lactobacillales*	*Streptococcaceae*	Unclassified	
	4435400	*Firmicutes*	*Clostridia*	*Clostridiales*	*Lachnospiraceae*	Unclassified	
	326936	*Firmicutes*	*Clostridia*	*Clostridiales*	*Lachnospiraceae*	*Blautia*	Unclassified
	185972	*Firmicutes*	*Clostridia*	*Clostridiales*	*Lachnospiraceae*	*Ruminococcus*	Unclassified
	187272	*Firmicutes*	*Clostridia*	*Clostridiales*		Unclassified	
	1132942	*Firmicutes*	*Clostridia*	*Clostridiales*		*Ruminococcus*	Unclassified
OTUs in RE and SE	4458700	*Firmicutes*	*Clostridia*	*Clostridiales*	*Lachnospiraceae*	*Ruminococcus*	Unclassified
	313037	*Firmicutes*	*Clostridia*	*Clostridiales*		Unclassified	
	181074	*Firmicutes*	*Erysipelotrichi*	*Erysipelotrichales*	*Erysipelotrichaceae*	*cc_115*	Unclassified

NSC shown rarity of an unclassified species of *Ruminococcus* (ID 182245) and overabundance of two unclassified species of *Rikenellaceae* (ID 4336943, 157573, respectively) in RC; Overabundance of ID 182245 in RE; Rarity of ID 182245 and overabundance of a different unclassified species of *Ruminococcus* (ID 185972) in SC; Overabundance of ID 182245 and a third species of unclassified *Ruminococcus* (ID 548503) in SE.

Table 5 summarized the results of the Venn Diagram/NSC analyses. *Eubacterium dolichum* was rare in RC but became overabundant when RES was fed the cholesterol diet (RE). An unclassified species of *Lactobacillaceae* was found in abundance in SC but the same species was rare in RE. On the other hand, two *Lactobacillus* species (also in the *Lactobacillaceae* Family) were only found in RC, and an unidentified *Lachnospiraceae* species was abundant in RE but rare in SC.

**Table 5:** **Summary of key OTUs characteristics generated by Venn diagram and NSC analysis**.

	**RC**	**SC**
Abundant+	Uncl. *Clostridia* ( 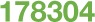 )	*Blautia producta* (158211)
		Uncl. *Coprococcus* (186319)
		Uncl. *Lachnospiraceae* (NCUR3)
		Uncl. *Ruminococcus* (185972)
		Uncl. *Lactobacillaceae* ( 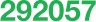 )
		Uncl. *Coprobacillaceae* (136526)
		Uncl. *Coprobacillaceae* ( 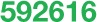 )
		***Bacteroidetes***
		Uncl. *Rikenellaceae* (4476780)
Abundant-	*Eubacterium dolichum* ( 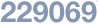 )	Uncl. *Clostridia* ( 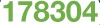 )
		Uncl. *Lachnospiraceae* ( 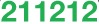 )
Unique	Uncl. *Lactobacillus* (137580)	Uncl. *Clostridia* (188057)
	*Lactobacillus reuteri* (137043)	
	**RE**	**SE**
Abundant+	*Eubacterium dolichum* ( 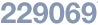 )	Uncl. *Ruminococcus* (548503)
	Uncl. *Lachnospiraceae* ( 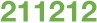 )	Uncl. *Coprococcus* (357765) Uncl. *Lachnospiraceae* (158971) Uncl. *Ruminococcaceae* (509101)
Abundant-	Uncl. *Coprobacillaceae* ( 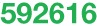 )	
	Uncl. *Lactobacillaceae* ( 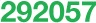 )	
Unique	Uncl. *Lachnospiraceae* (183867)	Uncl. *Ruminococcaceae* (228232)
	Uncl. *Lachnospiraceae* (2182669)	Uncl. *Ruminococcaceae* (189309)

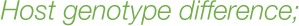

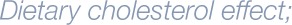

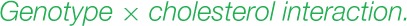

### Association of key bacteria species with plasma lipid parameters

There was a significant diet × genotype interaction in plasma Total Cholesterol (TC) (*P* < 0.006; Table 6), LDL (*P* < 0.004; Table 7) levels, and LDL/HDL ratio (*P* < 0.004 Table 8). SE was significantly higher in these parameters than the other three treatment groups.

**Table 6:** **Significant (*P* < 0.006) Diet × Genotype interaction in plasma total cholesterol level (mmol/L)**.

***N* = 16**	**Genotype**	
**Diet**	**RES**	**SUS**
Control	4.75±0.39^a^	5.29±0.46^a^
Cholesterol	14.10±1.07^a^	36.65±6.57^b^

Means followed by different letter superscripts are significantly different by Tukey's HSD.

**Table 7:** **Significant (*P* < 0.004) Diet × Genotype interaction in plasma LDL level (mmol/L)**.

***N* = 16**	**Genotype**	
**Diet**	**RES**	**SUS**
Control	1.04±0.06^a^	1.29±0.10^a^
Cholesterol	8.07±2.09^a^	32.03±6.42^b^

Means followed by different letter superscripts are significantly different by Tukey's HSD.

**Table 8:** **Significant (*P* < 0.004) Diet × Genotype interaction in plasma LDL/HDL ratio**.

***N* = 16**	**Genotype**	
**Diet**	**RES**	**SUS**
Control	0.34±0.02^a^	0.38±0.05^a^
Cholesterol	2.45±0.87^a^	8.52±1.48^b^

Means followed by different letter superscripts are significantly different by Tukey's HSD.

**Figure 7:**
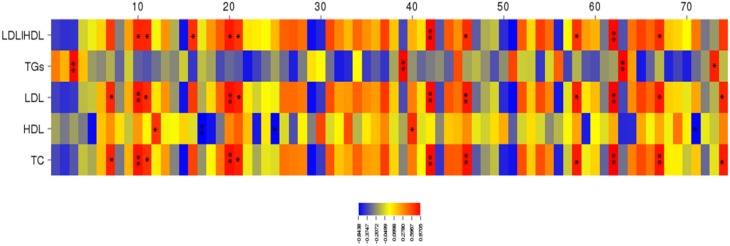
**Correlations heat map demonstrating the association between the abundances of different cecal microbial species and plasma lipid parameters**. Correlation coefficients are represented by color ranging from blue, negative correlation, to red, positive correlation. Significant correlations are noted by ^*^p < 0.05 and ^**^p < 0.01.

The abundance of four species of ceca bacteria in *Lachnospiraceae*, three species in *Ruminococcaceae*, and one species in *Coprobacillaceae* were positively correlated with the plasma TC, plasma LDL, and LDL/HDL ratio (Figure 7 and Table 9). The abundance of one species of *Lachnospiraceae* was positively correlated with plasma HDL while the abundance of two species of *Lachnospiraceae* and *Eubacterium dolichum* was negatively correlated with plasma HDL level. The abundance of four species (Unclassified *Rikenellaceae* (157573), Unclassified *Oscillospira* (366143), Unclassified *Ruminococcaceae* (295861), and Unclassified *Cloacamonaceae* (NCUR 160)) were found to be significantly and positively correlated with plasma triglycerides level due to a single outlier point and were eliminated.

**Table 9:** **Significant Pearson's Correlations^§^ between the abundance of cecal bacteria species and plasma lipid parameters**.

**OTUs**	**Total Chol**	**HDL**	**LDL**	**LDL/HDL**
***FIRMICUTES***				
***Lachnospiraceae***				
Uncl. R*uminococcus* (229097)	0.85^**^		0.85^**^	0.78^*^
Uncl. *Ruminococcus* (174695)	0.73^*^		0.74^*^	0.76^*^
Uncl. *Ruminococcus* (263138)		0.72^*^		
Uncl. *Ruminococcus* (130214)				0.74^*^
Uncl. *Ruminococcus* (130103)		−0.84^**^		
Uncl. *Ruminococcus* (548503)	0.88^**^		0.88^**^	0.83^**^
Uncl. *Ruminococcus* (191273)	0.80^*^		0.79^*^	0.71^*^
Uncl. *Blautia* (326936)	−0.80^**^			
***Ruminococcaceae***				
Unclassified (189309)	0.89^**^		0.98^**^	0.90^**^
Unclassified (566391)	0.85^**^		0.83^**^	0.75^*^
Unclassified (988932)	0.82^*^		0.80^*^	0.72^*^
***Clostridia***				
Unclassified (178304)	0.97^**^		0.95^**^	0.90^**^
***Erysipelotrichaceae***				
*Eubacterium dolichum* (229069)		−0.71^*^		
***Coprobacillaceae***				
Unclassified (NCUR 1)	0.84^**^		0.82^**^	0.74^*^

§ After elimination of significant correlations due to a single outlier;

* P < 0.05;

** P < 0.01.

## Discussion

We examined the cecal microbiota of 12 week old quail that had been fed their respective diets for at least 6 weeks. The cecal microbiota should be mature and stable by that time (Lu et al., 2003). Taxonomic analysis showed that composition of the quail's cecal microbiota at various levels is similar to that of human, mice, hamsters, chickens, emu, and Bobwhite quail (Eckburg et al., 2005; Ley et al., 2005; Turnbaugh et al., 2008; Karlsson et al., 2012; Bennett et al., 2013; Su et al., 2014; Videnska et al., 2014). At the Phylum level, *Bacteroidetes* and *Firmicutes* dominated the cecal microbiota, representing over 98% of all the sequences detected. An unusual feature of the quail cecal microbiota was that all (>99.9%) *Bacteroidetes* were of the Family *Rikenellaceae.* At the Family level, the predominant taxons were *Rikenellaceae, Lactobacillaceae, Streptococcaceae, Lachnospiraceae, Coprobacillaceae*, and *Erysipelotrichaceae*. At the Genus level, *Ruminococcus, Blautia, Coprococcus, Eubacterium* were abundant. While in most cases, it was not possible to identify the OTUs down to the species level because of high similarity of the 16S sequences among different bacterial species, we were able to identify a few OTUs down to the species level (e.g., see Table 4) because of their unique 16S sequences. We have BLAST searched these OTU sequences and found 100% sequence similarity with the species identified. Since even the closest species members of those bacteria have at least 5% sequence dissimilarity, we are confident that we have correct taxonomic identification for these OTUs.

### The effect of host genotype on cecal microbiota when birds were on control diet

The SUS and RES quail strains are a result of divergent selection from a common foundation population. The selection criteria being the highest and lowest atherosclerotic plaque scores, respectively, when the birds were fed a diet containing 1% cholesterol (w/w) (Shih et al., 1983). As a correlated response, we found that the selection not only modified the quail cecal environment to host a different cecal microbiome when the birds were fed a regular diet, but they also reacted differently when fed a high cholesterol diet. In birds, embryos develop in the egg outside the mother's body and parental influence should be minimum. In our study, the eggs of the four treatment groups were artificially incubated at the same time in close proximity in the same incubator. The birds of the two strains fed the same diet were raised in the same pen. The housing density was low enough to minimize any possibility of one strain dominating the other and affected their feed intake. This was confirmed by the fact that body weight and the mortality rate were not different between the two strains. The difference in cecal microbiota between RC and SC can therefore be attributed to host genetic differences in affecting the cecal environment.

While there was no significant difference in CHAO1 richness and Simpson diversity between RC and SC cecal microbiome, PCA analysis of cecal microbiota at the Phylum level detected two distinct *Firmicutes* communities between RC and SC. At the family level, RC was characterized by significant abundance of *Ruminococcaceae*. At the OTU level, 10 OTUs were unique to RC. Combining the results from the Venn diagram and NSC analyses, we concluded that RC hosted two unique species, *Lactobacillus reuteri* (ID 137043) and an unclassified *Lactobacillus* (ID 1375808), in significant abundance. In comparison with other treatment groups, RC has less abundance of *Eubacterium dolichum* (ID 229069) but more abundance in an unclassified species of *Clostridia* (ID 178304). *Ruminococcaceae* is one of the two most abundant families from the order *Clostridiales* found in the mammalian gut environment, and have been associated with the maintenance of gut health [38]. *Ruminococcaceae* is also the most common family of microbes in chicken cecum (Apajalahti and Kettunen, 2006; Torok et al., 2011). *L. reuteri* has been commonly used as a probiotic to suppress GI tract inflammation in human (Shornikova et al., 1997). Resting cells of this species convert glycerol into a potent, broad-spectrum antimicrobial substance termed reuterin (Axelsson et al., 1989). Dietary supplementation with *L. reuteri* ATCC 55730 resulted in significant colonization in the stomach, duodenum, and ileum of healthy humans, and this is associated with significant improvements of the immune response in the gastrointestinal mucosa (Valeur et al., 2004). Selection for resistance to diet induced atherosclerosis may have improved the general gut (cecum) health of RES quail.

At the family level, SC was characterized by abundance of *Coprobacillaceae*. Out of the six OTUs identified by Venn Diagram Analysis as unique in SC, only one was deemed significantly abundant by NSC analysis: an unclassified *Clostridia* species (ID 188057). Comparing with other treatment groups, SC has more abundance of four *Lachnospiraceae* species, including *Blautia producta* (ID 158211) and unclassified *Coprococcus* (ID 186319). SC also has more abundance of two unclassified *Coprobacillaceae* species (ID 136526 and 592616), an unclassified *Lactobacillaceae* species (ID 292057), and an unclassified *Rikenellaceae* (Bacteroidetes) species (ID 4476780). In the less abundant category, SC has an unclassified *Clostridia* (ID 178304) which was significantly more abundant in RC, and an unclassified Lachnospiraceae species (ID 211212). Although *Coprobacillaceae* is found in most of the microbiome data set of different hosts, it is not a well-studied Family and not much information is available about activities of members of this Family (Verbarg et al., 2014). *Lachnospiraceae* species are cellulose-degrading bacteria prevalent in bovine gut samples. They produce butyric acid to degrade plant fiber such as xylans. In human, butyrate arising from such microbial fermentation is important for the energy metabolism and normal development of colonic epithelial cells and has a mainly protective role (activating immune/inflammatory responses) in relation to colonic disease (Pryde et al., 2002; Maslowski et al., 2009; Vinolo et al., 2011). Mice precolonized with a murine *Lachnospiraceae* isolate had significantly decreased *Clostrium difficile* (a pathogen) colonization, lower intestinal cytotoxin levels and exhibited less severe clinical signs and colonic histopathology (Reeves et al., 2012). Many *Clostridia* species are toxigenic (Hatheway, 1990). The question remains whether the unclassified *Clostridia* species (ID 188057) that was only found in SC is pathogenic. Videnska et al. (2014) examined the succession and replacement of bacterial population in the cecum of laying hens at various ages and found that cecal microbiota in young chicks were dominated by *Firmicutes*, but as the hen became sexually mature and started egg production, a gradual succession of the representatives of *Firmicutes* and also their replacement with the representatives of *Bacteroidetes* was observed (Videnska et al., 2014). *Rikenellaceae* only started to colonize the cecum when the hen was in full egg production. Atherosclerosis is also accelerated by aging (Weingand et al., 1986; Clarkson et al., 1987; Collins et al., 2009). The hypothesis that selection for susceptibility to diet-induced atherosclerosis has also pre-maturely aged the cecal environment is worth testing.

Divergent selection for resistance and susceptibility to dietary cholesterol induced atherosclerosis has shifted the cecal microbiome of Japanese quail in different ways.

### The effect of dietary cholesterol on cecal microbiota

The diet we used for the study was a synthetic diet where all the dietary ingredients were known and standardized. The composition of the control and experimental diets was identical except for the added cholesterol and a small amount (0.02% w/w) of Cholic acid to aid the digestion and absorption of cholesterol. The difference in cecal microbiota between birds fed the control diet and the experimental diet can therefore be attributed to dietary cholesterol.

Dietary cholesterol has major effects on cecal microbiota in RES and SUS alike. At the Family level, birds on cholesterol diet had significantly more *Erysipelotrichaceae*, but significantly less unclassified *Bacilli* and unclassified *Clostridia* than birds on control diet. At the genus level, birds fed the cholesterol diet had significantly less abundance of unclassified *Ruminococcus*. All members of the *Erysipelotrichaceae* Family were associated with one or several hosts including mammals, birds, fish and marine invertebrates, and most members were found as opportunistic pathogens affecting various parts of the body (Verbarg et al., 2014). Dietary cholesterol has reduced the abundance of *Ruminococcus* and facilitated the abundance of opportunistic pathogens in the quail ceca and may have increased the risk of assaults by these opportunistic pathogens.

### The effect of genotype × diet interaction on cecal microbiota

When SUS and RES were put on a high cholesterol diet, they also reacted differently, in terms of the microbiota that they were hosting, to the dietary cholesterol. CHAO1 richness of cecal microbiome in RE was significantly reduced when compared with RC, but CHAO1 richness of cecal microbiome in SUS was not affected by diet.

At the family level, cecal microbiota in RC was characterized by the relative abundance of *Ruminococcaceae*, unclassified *Clostridiales, Bacilli* and *Clostridia*, and RE by abundance of *Erysipelotrichaceae*. RC had significantly more *Ruminococcaceae* than RE. SC was characterized by abundance of *Coprobacillaceae*, but SE was characterized by abundance of *Lachnospiraceae*. The abundance of *Ruminococcaceae* was not affected by diet in SUS. At the OTU level, *Eubacterium dolichum* was rare in RC but became overabundant in RE. Both SC and SE showed rarity of *E*. *dolichum*.

When mice were fed a “Western diet” which was high in fat and cholesterol, the overall diversity of their gut microbiota dropped significantly due to a bloom of a class of *Firmicutes* called *Mollicutes*, a member of which is *E*. *dolichum* (Turnbaugh et al., 2008). *E. dolichum* has a number of genomic features that could promote their own fitness in competition with other microbes in the cecal nutrient metabolic milieu created by the host's consumption of the Western diet (Turnbaugh et al., 2008). Their abundance is associated with obesity in mice. A similar situation may have occurred in RE in their reaction to dietary cholesterol. SE has abundance of *Lachnospiraceae*. At the same time, their abundance of *Ruminococcaceae* was not compromised by dietary cholesterol. *Lachnospiraceae* and *Ruminococcaceae* have been associated with the maintenance of gut health (Place et al., 2005; Huws et al., 2011; Vinolo et al., 2011; Reeves et al., 2012; Biddle et al., 2013; Greer et al., 2013). These two families are specialists for degrading cellulose and hemicellulose components of plant materials which are fermented and converted into short chain fatty acids (SCFAs) be absorbed and used by the host (Biddle et al., 2013). SCFAs have an important roles in maintaining intestinal homeostasis (Pryde et al., 2002; Place et al., 2005; Cotta and Forster, 2006; Wong et al., 2006; Greer et al., 2013). The results from our study seem to indicate that the divergent selection for susceptibility/resistant to diet induced atherosclerosis has adversely affected the cecal health of RE but not SE, via their cecal microbiome. Whether this change in the cecal environment has effects on the metabolism and absorption of dietary cholesterol remains to be studied.

### Cecal microbiota and atherosclerosis

Recently there has been a flourish of studies on the relationship between gut microbiota and cardiovascular diseases in human and in animal models. In human, about 50% of dietary cholesterol is absorbed in the duodenum. All cholesterol arriving in the large intestine can be metabolized by *Eubacterium* bacteria to coprostanol and minor amounts of coprostanone (Macdonald et al., 1983). Coprostanol, unlike cholesterol, is poorly absorbed by the human intestine and hence, conversion of cholesterol to coprostanol might be a way to lower serum cholesterol in human and rodents (Sekimoto et al., 1983; Li, 1995; Stepankova et al., 2010). However, feeding *Eubacterium coprostanoligenes* to laying hens failed to lower plasma cholesterol (Li et al., 1996). In our study, *Eubacterium dolichum* was found in abundance in the RE cecum but not in SE. However, the ability of *E. dolichum* to convert cholesterol to coprostanol has not been demonstrated although we have found a significant but negative correlation of *E. dolichum* abundance with plasma HDL level. On the other hand, the primary cholesterol absorption sites are in the small intestine and it will be worthwhile to examine the microbiota in duodenum and ileum (S Liu and KM Cheng, study in progress).

In human and mice, intestinal microbes (*Clostridium, Peptostreptococcaceae, Tenerites*, and *Clostridiaceae*) can catabolize choline and L-carnitine to gaseous trimethylamine (TMA) (Al-Waiz et al., 1992; Koeth et al., 2013) which can be efficiently absorbed and metabolized by hepatic enzymes, Flavin monooxygenase 3 (FMO3), to form TMAO, an oxidized product of TMA (Cashman et al., 2003). TMAO promotes atherosclerosis by up-regulation of macrophage scavenger receptors (Wang et al., 2011) and by down-regulating genes involved in reverse cholesterol transportation (Koeth et al., 2013). The dietary sources of choline are foods such as eggs, milk, red meat, liver, shell fish, and fish, which are rich in lecithin (Wang et al., 2011). The dietary source of L-carnitine is red meat (Koeth et al., 2013). None of these food items are in the natural diet of Japanese quail. The synthetic diets that we have prepared for the study are also very low in choline (0.04% w/w of choline chloride) and L-carnitine. The atherosclerosis we found in the SE may not be promoted through this pathway but Shih et al. (1983) and Godin et al. (2003) reported that after cholesterol feeding, plasma cholesterol levels remained high for a significantly longer time in SE than in RE (Shih et al., 1983). Li et al. (2012) also found down regulation of some of the cholesterol transport genes in the SE liver (Li et al., 2012). It will be worthwhile to examine the expression of hepatic *FMO3* and RCT associated genes which can be regulated by TMAO, in the SE compared with RE (JE Kim and KM Cheng, study in progress).

It has been proposed that bacterial lipopolysaccharide (LPS), a constituent of Gram negative bacteria present in the gut microbiota, can be transported from the intestine to target tissue and combine with CD14 and the toll-like receptor 4 (TLR4) at the surface of innate immune cells such as macrophages. Such “metabolic endotoxemia” can trigger the secretion of proinflammatory cytokines. Efflux of cholesterol from vessel wall macrophages is believed to be a critical first step by which RCT protects against atherosclerosis. TLR4 inhibits RCT and thus may modulate cholesterol metabolism. These findings suggest that gut microbiota may be important for RCT but it is not clear whether gut microbiota contributes to atherosclerosis through this pathway (Caesar et al., 2010). In our study, RE is the only group that has abundant gram negative bacteria (*E. dolichum*) in their cecal microbiota and yet they were resistant to diet induced atherosclerosis. On the other hand, the abundance of several gram positive species (*Lachnospiraceae* and *Ruminococcaceae*) was positively correlated with plasma TG and plasma LDL levels. The hypothesis that selection for susceptibility to diet induced atherosclerosis in Japanese quail has also shifted their gut microbiota to enhance the metabolism and absorption of cholesterol remains to be tested.

Several animal models have been developed for studying atherosclerosis, but each has advantages and limitations (Jokinen et al., 1985; Getz and Reardon, 2012; Kapourchali et al., 2014). The Japanese quail model may have advantages over others because quail are naturally deficient in apolipoprotein E. When fed a high cholesterol diet, males of the SUS strain developed lesions exhibiting structural features (e.g., focal hemorrhage, calcification and fibrosis) that closely resemble those in the human disorder (Shih et al., 1983). Atherogenic diets increase the LDL and VLDL fraction of cholesterol with minimal effects on HDL, thus facilitating the study of metabolism and transport of cholesterol in relationship to atherosclerosis (Li et al., 2012). Our study of cecal microbiota in these quail has demonstrated that selection for susceptibility/resistance to diet induced atherosclerosis has also affected the quail's cecal environment to host distinctly different cecal microbiome. Moreover, the SUS and RES quail also reacted differently, in terms of the cecal microbiota that they are hosting, to dietary cholesterol. Our study allowed us to raise new questions about the relationship between gut microbiota and cholesterol metabolism.

## Author contributions

This manuscript is an extension of the thesis research carried out by SL. DB provided expertise in quail nutrition and gut microbiota. HT provided expertise in Bioinformatics. JK provided expertise in DNA extraction. FL provided expertise and laboratory facilities for pyrosequencing. HZ was SL's thesis supervisor at CAAS. KC was the thesis research supervisor and provided expertise in genetics, cholesterol metabolism and atherosclerosis, and experimental design.

### Conflict of interest statement

The authors declare that the research was conducted in the absence of any commercial or financial relationships that could be construed as a potential conflict of interest.

## Abbreviations

FMO3: Flavin monooxygenase 3
LPS: lipopolysaccharide
OUTs: operational taxonomic units
PCA: Principal Components Analysis
RC: atherosclerosis-resistant quail on control diet
RCT: reverse cholesterol transport
RE: atherosclerosis-resistant quail on cholesterol diet
RES: atherosclerosis-resistant quail
SC: atherosclerosis-susceptible quail on control diet
SE: atherosclerosis-susceptible quail on cholesterol diet
SUS: atherosclerosis-susceptible quail
TG: triglycerides
TLR4: toll-like receptor 4
TMA: trimethylamine
TMAO: trimethylamine N-oxide.
